# An atypical 7Q11.2-Q21.11 deletion in a Williams-Beuren syndome patient

**DOI:** 10.1186/1687-9856-2013-S1-P161

**Published:** 2013-10-03

**Authors:** Wei Lu, Yi Yang, Xiao-hong Guo, Lin Yang, Hui-jun Wang, Ren-chao Liu, Yu An, Fei-hong Luo

**Affiliations:** 1 Department of Endocrine and Inherited Metabolic Diseases, Children’s Hospital of Fudan University, Shanghai, China; 2 Pediatric Research Institute, Children’s Hospital of Fudan University, Shanghai, China

## Aims

Williams–Beuren syndrome (WBS; OMIM no. 194050) is a multisystemic neurodevelopmental disorder caused by a hemizygous deletion of 1.55Mb on chromosome 7q11.23 spanning 28 genes. Here we report a patient showing mild WBS physical phenotype, who carries a longer 21Mb atypical deletion.

## Methods

Genomic DNA from the proband was extracted from peripheral blood leukocyte. Karyotype analysis was performed on metaphase cells. Array-based comparative genomic hybridization of DNA from the patient’s peripheral blood lymphocytes was performed.

## Results

The proband, a female neonate, is the first child of healthy nonconsanguineous Chinese parents. She was born by uterine-incision delivery with intrauterine distress after 41 weeks of gestation. Her birth weight was 2.4 kg. She showed an distinctive facies including broad brow, periorbital fullness, epicanthal folds, short nose, long philtrum, small jaw and prominent earlobes. The cardiology ultrasound examination showed open foramen ovale without elastin arteriopathy such as supravalvular aortic stenosis, pulmonic stenosis. Her abdominal ultrasound examination showed right Duplicated kidneys. Her Karyotyping was 46, XX, del(7)(q11.1q11.23). We then performed array CGH for this patient and confirmed the deletion region of 21Mb from 7q11.2 to 7q21.11.

## Conclusion

A deletion of the 7q11.23 chromosomal region is found in approximately 90-95% of the clinically typical WBS patients but in a lower percentage of atypical cases. The commonly deleted chromosomal region has a size of approximately 1.5 Mb. However, smaller or longer deletions have also been described in atypical WBS patients. Array CGH analysis can be performed to make sure the location and size of microdeletion and confirmed the diagnosis of patients with mild or atypical physical phenotypes.

